# Appropriate Total cholesterol cut-offs for detection of abnormal LDL cholesterol and non-HDL cholesterol among low cardiovascular risk population

**DOI:** 10.1186/s12944-019-0975-x

**Published:** 2019-01-26

**Authors:** Nopakoon Nantsupawat, Apaputch Booncharoen, Anawat Wisetborisut, Wichuda Jiraporncharoen, Kanokporn Pinyopornpanish, Lalita Chutarattanakul, Chaisiri Angkurawaranon

**Affiliations:** Department of Family Medicine, Faculty of Medicine, Chiang Mai University, 110 Intawaroros Road, Suthep, Muang, Chiang Mai, 50200 Thailand

**Keywords:** Total cholesterol, low-density lipoprotein cholesterol, Non-high density lipoprotein cholesterol, Diagnostic test, Thailand

## Abstract

**Background:**

Current guidelines suggest screening for dyslipidemia in early adulthood. In Thailand, a screening total cholesterol level is most commonly used potentially due to the costs of the test. However, the appropriate TC cut-off point that correlates with elevated low-density lipoprotein cholesterol (LDL-C) and non-high-density lipoprotein cholesterol (Non-HDL-C) levels for the low cardiovascular risk younger population have not been examined extensively in the literature.

**Methods:**

This study identified 1754 subjects with low cardiovascular risk**.** All participants had a physical examination and a venous blood sample sent for laboratory assessment of fasting blood glucose, TC, LDL-C, HDL-C levels. A non-HDL-C level for everyone was calculated by subtracting HDL-C levels from their total cholesterol levels. Sensitivity and specificity of different TC cutoff points in detection of abnormal LDL-C levels (≥ 130 mg/dL and ≥ 160 mg/dL) and abnormal non-HDL-C levels (≥ 160 mg/dL and ≥ 190 mg/dL) were calculated. Receiver operating characteristics (ROC) curve analysis was used to evaluate the predictive utility of TC for the abnormal LDL-C and abnormal non-HDL-C levels.

**Results:**

The conventional range TC cut off point, between 200 to 240, had varying diagnostic properties for detection of elevated LDL-C and Non-HDL-C within this low risk population. A TC cut off point 210 would have a sensitivity of 70% and specificity of 92.5% for detection of LDL-C **≥** 130 and a sensitivity of 96.7% and specificity of 85.6% for identifying those with Non-HDL-C ≥ 160. The TC cut off point of 230 had a sensitivity of 74.9% and specificity of 92.0% in identifying those with LDL-C ≥ 160 and a sensitivity of 98.6% and specificity of 89.8% in detection of non-HDL-C ≥ 190.

**Conclusions:**

Early screening for dyslipidemia in young adults is suggested by many guidelines. This population is likely to be those with lower cardiovascular risk and may needed to have repeated screening over time. Screening using TC with appropriate a cut off points may be a more cost-effective screening test in settings with limited resources, coverage and accessibility.

## Background

Cardiovascular disease is a significant health problem as one of the leading causes of disability and death around the world [1–3]. The development of cardiovascular disease associated with abnormal cholesterol levels, consisting of high levels of serum total cholesterol (TC), high levels of low-density lipoprotein cholesterol (LDL-C), and low levels of high-density lipoprotein cholesterol (HDL-C) are established risk factors of cardiovascular disease [4–9]. It is estimated that 11.9% or 28.5 million adults age 20 and above have abnormal TC levels [2].

Current guidelines suggest screening for dyslipidemia in early adulthood. The ATP-III, U.S. Preventive Services Task Force, the American College of Cardiology/American Heart Association (ACC/AHA), and the American Association of Clinical Endocrinologists (AACE) 2017 suggests screening for abnormal lipid profiles in adults over 20 years of age [10–12]. The Thai 2016 Clinical Practice Guidelines on Pharmacologic Therapy of Dyslipidemia for Atherosclerotic Cardiovascular Disease (ASCVD) Prevention also recommended screening young adults age 21 and older for lipid disorders [13]. Detection of younger adults with lipid disorders could enable implementation of management strategies such as lifestyle modification or medications that could prevent negative cardiovascular outcomes or decrease risks of future cardiovascular events.

However, the implementation and access to screening among younger adults, who are more likely to have lower cardiovascular risk than older adults, varied by settings [14]. Access to screening for dyslipidemia in Thailand depended on age, health care plans, health insurance and financial status [15]. The Universal Coverage Scheme, covering approximately 75% (around 47 million people) of the entire population, does not include screening for dyslipidemia in their package [16]. For individuals with the Thai Social Security Scheme, only screening TC and HDL-C at age 20 years and older is available. For Thai adults under the government health care welfare right (government employees) TC and triglyceride (TG) screening starts at the age of 35. In the absence of any underlying risk factors or diseases, most Thai people may need to pay out of pocket for dyslipidemia screening.

As demonstrated in Thailand, TC is the choice for screening potentially due to the costs of the test. The price of TC test is around 60 baht (1.84 USD). This is two times lower than price of the LDL-C test which is 120 baht (3.67 USD) in Thailand. TC may be a more cost-effective screening test especially since testing may need to begin at younger ages and repeated throughout adulthood. The current appropriate TC cutoff to determine whether patients need further investigation and assessment is between 200 and 240 mg/dL [1, 17, 18]. However, the appropriate cut-off point for the younger population who may have low cardiovascular risk have not been examined extensively in the literature. A recent study suggests that a TC cut of point of between 200 and 240 may not be appropriate in identifying high LDL-C levels in apparently healthy people [19].

In clinical practice, LDL-C is used as a marker for diagnosis and treatment control and currently non-high-density lipoprotein cholesterol (non-HDL-C) could be a better marker for management of dyslipidemia [20, 21]. Thus, the study aimed to identify appropriate TC cutoff points that correspond to abnormal LDL-C and abnormal Non-HDL-C among a younger population with low cardiovascular risk. This information may be useful in settings where TC levels are considered as a screening test in younger populations as in Thailand.

## Methods

This was a retrospective study, utilized data from a non-communicable disease survey among health care workers in 2013. The detailed description of the study has been published [22]. In summary, 3204 participants (59.7% response rate) were interviewed according to the WHO STEPS survey [23]. Participants had a physical examination and venous blood sample sent for laboratory assessment of fasting blood glucose, TC, LDL-C, HDL-C and TG levels. A non-HDL-C level for each individual was calculated by subtracting HDL-C levels from the total cholesterol levels.

### Identification of participants with low cardiovascular risk

**Fig. 1:**
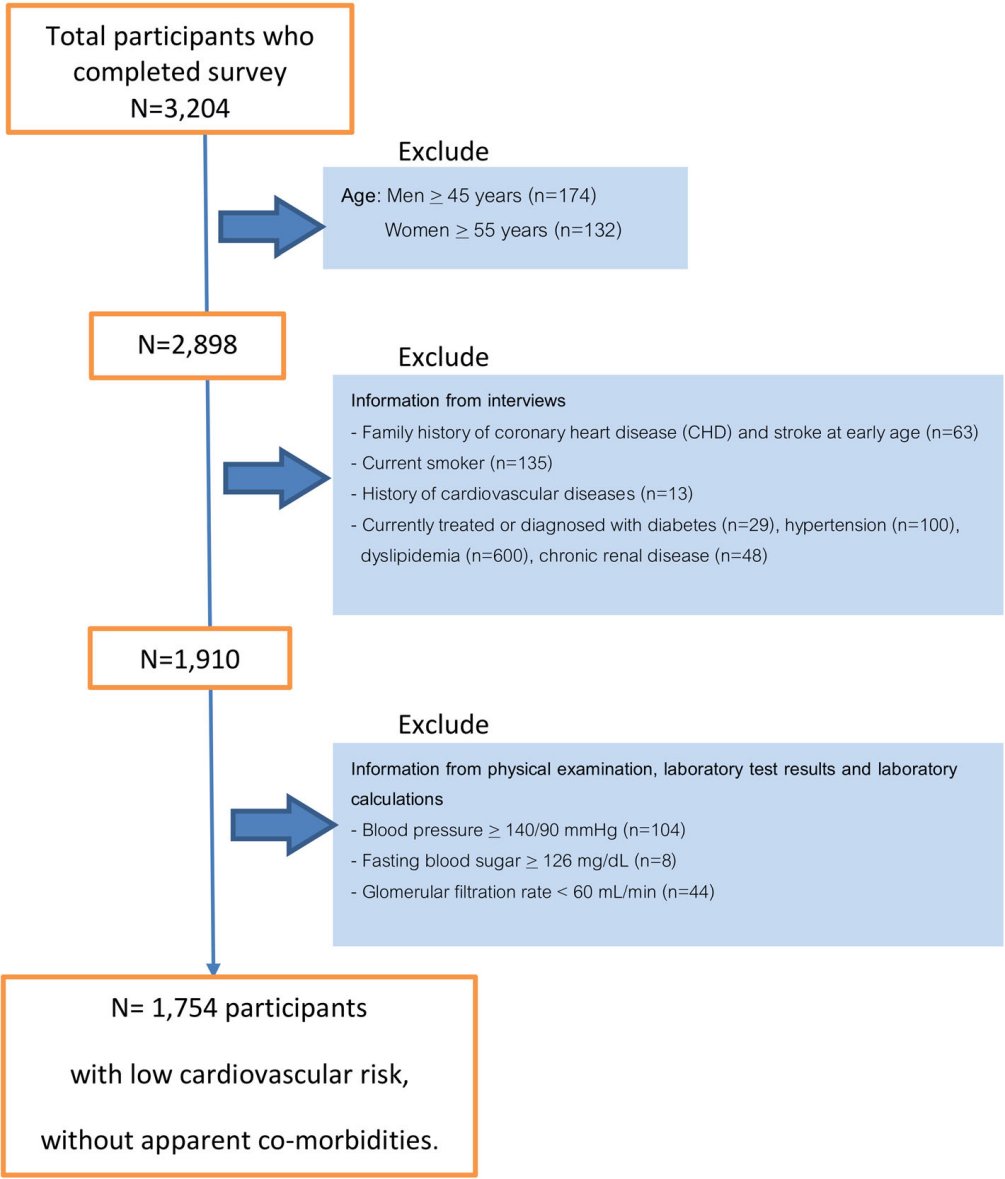
Identification of low cardiovascular risk participants

We identified participants with low cardiovascular risks based on their prior history as well as from their physical examination and laboratory results (Fig. 1). Participants were excluded from the analyses if they had any of the following cardiovascular risk factors:

1. History of cardiovascular disease (MI, stable or unstable angina, coronary or other arterial revascularization, stroke, transient ischemic attack (TIA), or peripheral arterial disease)
2. Age of men ≥45 years or women ≥55 years
3. Family history of coronary heart disease (CHD) and stroke at early age
4. Current smoker
5. History of or currently taking medications for the following conditions for the following conditions: diabetes, hypertension, dyslipidemia, or chronic renal disease
6. Elevated measurements: blood pressure ≥ 140/90 mmHg, fasting glucose ≥126 mg/dL or glomerular filtration rate (GFR) < 60 mL/min.

### Dyslipidemia cut off points using LDL-C and non-HDL-C

We identify two values of LDL-C cutoffs and two values of Non-HDL-C cutoffs according to the potential goal of screening programs. We proposed that if the aim of a screening was to identify those who may benefit from early lifestyle intervention, LDL-C ≥ 130 mg/dL or non-HDL-C ≥ 160 would be appropriate [12]. However, if the aim of a screening was to identify those with greater need for medication and more intensive intervention, an LDL-C ≥ 160 mg/dL or Non-HDL-C ≥ 190 mg/dL would be appropriate [24].

### Analysis plan

Stratified by sex, descriptive statistics were used to describe the sample. The sensitivity and specificity of different TC cutoff point in detection of abnormal LDL-C levels (≥130 mg/dL and ≥ 160 mg/dL) and abnormal non-HDL-C levels (≥ 160 mg/dL and ≥ 190 mg/dL) were calculated. Receiver operating characteristics (ROC) curve analysis was used to evaluate the predictive utility of TC for the abnormal LDL-C level and abnormal non-HDL-C level. Youden index was used to identify the optimal TC cut-off points. All analyses were performed using STATA version 15.0.

## Results

### Characteristics of the subjects

One thousand seven hundred fifty-four subjects (Fig. 1) were identified as having low cardiovascular risk factors, 197 men and 1557 women. The mean age was 32.9 years (sd 5.9) for men and 35.6 years (sd 9.1) for women. The baseline characteristics reflected low cardiovascular risks within the sample (Table 1). The average body mass index was 23.9 kg/m2 (sd 3.5) for men and 22.1 kg/m2 (sd 3.5) for women. The average fasting blood glucose level was at 87.1 mg/dl (sd 8.7). The mean systolic and diastolic blood pressure for men was 119.5 mmHg (sd 9.2) and 75.0 mmHg (sd 6.9) respectively. For women, the mean systolic and diastolic blood pressure was 108.4 mmHg (sd 10.1) and 68.8 mmHg (sd 8.2) respectively. Among this sample with low cardiovascular risks, about 46.2% had a total cholesterol of at least 200 mg/dL. The proportion with a LDL-C cholesterol level of at least 130 mm/dL was 42.7% and the proportion with non-HDL-C level of at least 160 mm/dL was 24.2%. The average TG level was 118.8 mg/dL (sd 71.1) for men and 77.2 mg/dL (sd 46.9) for women. The average HDL-C level was 52.7 mm/dL (sd 11.5) for men and 60.6 (12.2) for women (Table 2).

**Table 1:** Sample characteristics

	Total (*n* = 1754)	Female (*n* = 1557)	Male (*n* = 197)
Mean age in year (sd)	35.3 (8.9)	35.6 (9.1)	32.9 (5.9)
Mean BMI in kg/m2 (sd)	22.3 (3.6)	22.1 (3.5)	23.9 (3.5)
Mean waist circumference in cm (sd)	71.9 (9.3)	70.8 (8.7)	80.6 (9.7)
Mean glucose in mg/dl (sd)	87.1 (8.7)	86.7	89.8 (8.6)
Mean SBP in mmHg (sd)	109.7 (10.6)	108.4 (10.1)	119.5 (9.2)
Mean DBP in mmHg (sd)	69.5 (8.3)	68.8 (8.2)	75.0 (6.9)

**Table 2:** Lipid profile of participants

	Total (*n* = 1754)	Female (*n* = 1557)	Male (*n* = 197)
Mean TC (SD)	198.6 (35.7)	197.8 (35.1)	205.1 (39.7)
Proportion with TC > 240	12.4	11.0	23.4
Proportion with TC > 200	46.2	45.3	53.3
Mean LDL-c (sd)	126.1 (32.1)	124.9 (31.4)	134.9 (35.8)
Proportion with LDL > 160	14.1	12.5	26.9
Proportion with LDL > 130	42.7	41.4	52.8
Mean non-HDL-c (sd)	138.9 (34.1)	137.1 (33.2)	152.4 (37.9)
Proportion with non-HDL > 190	8.1	6.6	20.3
Proportion with non-HDL > 160	24.2	22.0	41.6
Mean HDL (sd)	59.7 (12.4)	60.6 (12.2)	52.7 (11.5)
Mean TG (sd)	81.9 (51.9)	77.2 (46.9)	118.8 (71.1)

### Total TC cut off points for detection of abnormal LDL-C level

The conventional range TC cut off point, between 200 to 240 had varying diagnostic properties within this low risk population for identifying participant with LDL-C ≥ 130 mg/dL. A TC cut off point of 200 correctly classified 83.3% of participants. The sensitivity and specificity of this cutoff point was 84.5 and 82.4% respectively. Using TC of 240 as the cutoff point, only classified 69% of the participants correctly. The sensitivity and specificity for this cutoff point of 240 was 22.2 and 99.4% respectively (Table 3).

**Table 3:** Total cholesterol cut off point for detection of elevated LDL cholesterol

	LDL 130				LDL 160			
TC cut-off point	Sensitivity	Specificity	Correctly classify	Likelihood ratio positive	Sensitivity	Specificity	Correctly classify	Likelihood ratio positive
≥180	97.3	52.6	71.7	2.05	99.6	36.4	45.3	1.56
≥190	93.3	69.6	79.8	3.07	99.2	49.6	56.6	1.97
≥200	**84.5**	**82.4**	**83.3**	**4.80**	98.4	62.4	67.5	2.61
≥210	70.0	92.5	82.9	9.38	**95.6**	**75.9**	**87.7**	**3.97**
≥220	54.2	96.7	78.6	16.5	87.5	85.2	85.5	5.91
≥230	38.7	98.5	73.0	25.9	74.9	92.0	89.6	9.41
≥240	28.2	99.4	69.0	47.2	64.4	96.1	91.7	16.7
≥250	18.8	99.9	65.3	189.2	49.8	98.7	91.9	39.5
≥260	12.4	99.9	62.5	124.8	33.6	99.3	90.0	46.0

For identifying those with LDL-C ≥ 160 mg/dL, the TC cutoff point of 200 only correctly classified 67.5% of participants with high sensitivity (98.4%) but low specificity (62.4%). The TC cutoff point of 240 correctly classified 91.7% of the participants with a sensitivity of 64.4% and specificity of 96.1% (Table 3).

### Total cholesterol (TC) cut off points for detection of abnormal non-HDL-C level

Similar to the detection of abnormal LDL-C levels, the conventional range TC cutoff point had varying diagnostic properties within this low risk population. For identifying those with non-HDL-C ≥ 160 mg/dL, the TC cutoff point of 200 correctly classified 78.0% of participants. While the sensitivity of this cutoff point was 100% and specificity was 71.0%. Using TC of 240 as the cutoff point, it only classified 87.3% of the participants correctly. The sensitivity and specificity for this cutoff was 49.3 and 99.4% respectively (Table 4).

**Table 4:** Total cholesterol cut off point for detection of elevated Non-HDL cholesterol

	Non-HDL 160				Non HDL 190			
TC cut-off point	Sensitivity	Specificity	Correctly classify	Likelihood ratio positive	Sensitivity	Specificity	Correctly classify	Likelihood ratio positive
≥180	100	41.3	55.5	1.70	100	34.1	39.4	1.51
≥190	100	56.4	66.9	2.29	100	46.6	50.9	1.87
≥200	100	71.0	78.0	3.45	100	58.6	62.0	2.41
≥210	**96.7**	**85.6**	**88.4**	**6.80**	100	71.7	74.0	3.53
≥220	84.2	93.8	91.5	13.6	100	81.6	83.1	5.44
≥230	64.9	97.7	89.8	28.7	98.6	89.8	90.5	9.68
≥240	49.3	99.4	87.3	81.9	**97.2**	**95.2**	**95.3**	**20.1**
≥250	33.0	99.8	83.7	219.6	78.3	98.1	96.5	42.0
≥260	21.7	99.8	81.0	144.3	59.4	99.4	96.2	106.4

For non-HDL-C ≥ 190 mg/dL, the TC cutoff point of 200 only correctly classified 62.0% of participants with high sensitivity (100%) but low specificity (58.6). The TC cutoff point of 240 correctly classified 95.3 of the participants with a sensitivity of 97.2% and specificity of 95.2%. (Table 4).

### Potential optimal TC cutoff points

**Fig. 2:**
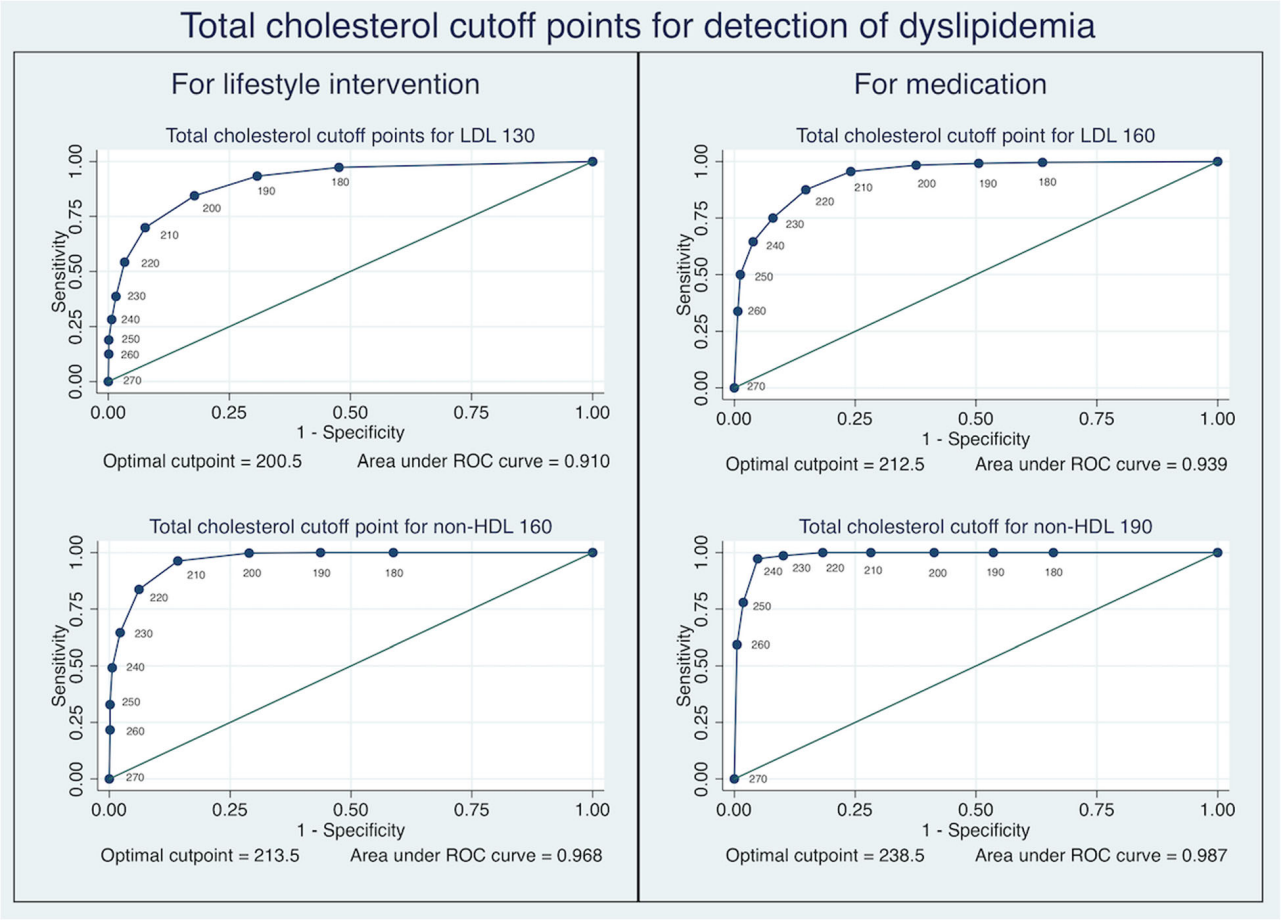
Total Cholesterol Cut-Offs for Detection of Abnormal LDL-C Cholesterol and Non-HDL-C Cholesterol Among Low Cardiovascular Risk Population

Classified by the potential purpose of a screening program, our study suggests that a total cholesterol cutoff point of 210 may be a useful cutoff for detection of abnormal lipids levels (LDL-C ≥ 130 and non-HDL-C ≥ 160) for which lifestyle interventions may need to be considered (Fig. 2). A TC cutoff point 210 would have a sensitivity of 70% and specificity of 92.5% for detection of LDL-C ≥ 130 and a sensitivity of 96.7% and specificity of 85.6% for identifying those with Non-HDL-C ≥ 160.

For programs aimed at identifying those at higher risk (LDL-C ≥ 160 and non-HDL-C ≥ 190), the TC cutoff point of 230 may be useful (Fig. 2). This cutoff point has a sensitivity of 74.9% and specificity of 92.0% in identifying those with LDL-C ≥ 160 and a sensitivity of 98.6% and specificity of 89.8% in detection of non-HDL-C ≥ 190.

## Discussion

In our study, we analyzed the relationships between different values of TC, LDL-C, and non-HDL-C in a population with low cardiovascular risk. The data is in line with the appropriate ranges suggested by the World Health Organization for detection dyslipidemia based on TC levels, which is between 200 and 240 mm/dL. Our study added more information that if the objective of screening is to provide early lifestyle intervention, a TC cut of point of 210 may be suitable which gives a relatively high sensitivity and specificity for both high LDL-C ≥ 130 and high non-HDL-C ≥ 160. However, if the main objective in screening this relatively low risk group is to identify those with greater need for intervention and medication, a TC cut of point of 230 gives a high sensitivity and specificity for both high LDL-C ≥ 160 and non-HDL-C ≥ 190.

The optimal TC levels suggested differed from Zhang et al. [19] which suggested an optimal threshold at 182.5 mg/dL for detection of the abnormal LDL-C level (LDL-C ≥ 130 mg/dl) in apparently healthy populations. However, the study by Zhang only had access to laboratory results and did not have access to past medical history and physical examination, which may not be generalizable to healthy populations or those with low cardiovascular risks. Our suggested cut off point of 210 is line with other previous studies [25, 26] that showed TC cutoff range from 200 to 210 mg/dL was appropriate for early intervention while the TC cutoff point of 230 to identify those at high risk is also supported by the literature in a large population study with 10-year follow up [27, 28]. These studies demonstrated that a higher risk of ischemic heart disease was found in the high cholesterol group with a TC level ≥ 240 mg/dL.

While TC levels may not reflect the true risk of cardiovascular risk from dyslipidemia, however, implementation as a screening tool in low to medium income countries could help reduce the expense of screening and increase accessibility at a population level [19]. As previously stated, the price of TC test is two-times lower than price of LDL-C. Therefore, difference in the costs would be almost 45 million US dollars for Thailand if TC was used rather than LDL-C for those between 21 and 45 years (approximately 24,615,016 individuals) [1].

There were some limitations to this study. The use of interviews to obtain some cardiovascular risk factors such as smoking could be prone to social desirability bias and at risk of some misclassification. However, the interviews were conducted by those not working in the hospital to minimize this issue. The study was a single-center study with a large proportion of female subjects but sensitivity analysis stratified by gender did not yield materially different conclusions (data not shown). We did not consider HDL-C levels as part of the initial criteria for identifying those with low cardiovascular risk factors. This is because we wanted to replicate scenarios where low risk participants were coming for a screening. Thus, it is possible that we may not have entirely captured the low cardiovascular risk population. The results from our study may not be generalizable to low cardiovascular risk populations in other regions as factors influencing cholesterol levels may vary. For example, in regions where high nutraceuticals and functional food ingredients are more common, on average, lipid levels may lower [29]. However, cutoff points for elevated cholesterol generally do not vary by populations and regions. Similar studies conducted in different settings may be needed to validate the proposed cutoff points in this study.

## Conclusions

Early screening for dyslipidemia in young adults is suggested by many guidelines. This population is likely to be those with lower cardiovascular risk and may needed to have repeated screening over time. Screening using TC with appropriate a cutoff points may be a more cost-effective screening test in settings were resources, coverage and accessibility are limited. A TC cutoff point of 210 may be suitable to identify those who may need further investigation and early lifestyle intervention. However, if the main objective on screening in this relatively low risk group is to identify those with greater need for intervention and medication, a TC point of 230 may be a more appropriate cutoff point.

## Abbreviations

AACD: American Association of Clinical Endocrinologists
ASCVD: Atherosclerotic Cardiovascular Disease
HDL-C: high-density lipoprotein cholesterol
LDL-C: Low-density lipoprotein cholesterol
Non-HDL-C: Non-high-density lipoprotein cholesterol
TC: Total cholesterol
TG: Triglyceride
